# An Overview of the Electron-Transfer Proteins That Activate Alkane Monooxygenase (AlkB)

**DOI:** 10.3389/fmicb.2022.845551

**Published:** 2022-02-28

**Authors:** Shoshana C. Williams, Rachel Narehood Austin

**Affiliations:** ^1^Department of Chemistry, Stanford University, Stanford, CA, United States; ^2^Department of Chemistry, Barnard College, Columbia University, New York City, NY, United States

**Keywords:** alkane monooxygenase, AlkB, rubredoxin, fusion-protein, hydrocarbon oxidation

## Abstract

Alkane-oxidizing enzymes play an important role in the global carbon cycle. Alkane monooxygenase (AlkB) oxidizes most of the medium-chain length alkanes in the environment. The first AlkB identified was from *P. putida* GPo1 (initially known as *P. oleovorans*) in the early 1970s, and it continues to be the family member about which the most is known. This AlkB is found as part of the OCT operon, in which all of the key proteins required for growth on alkanes are present. The AlkB catalytic cycle requires that the diiron active site be reduced. In *P. putida* GPo1, electrons originate from NADH and arrive at AlkB *via* the intermediacy of a flavin reductase and an iron–sulfur protein (a rubredoxin). In this Mini Review, we will review what is known about the canonical arrangement of electron-transfer proteins that activate AlkB and, more importantly, point to several other arrangements that are possible. These other arrangements include the presence of a simpler rubredoxin than what is found in the canonical arrangement, as well as two other classes of AlkBs with fused electron-transfer partners. In one class, a rubredoxin is fused to the hydroxylase and in another less well-explored class, a ferredoxin reductase and a ferredoxin are fused to the hydroxylase. We review what is known about the biochemistry of these electron-transfer proteins, speculate on the biological significance of this diversity, and point to key questions for future research.

## Introduction

Alkane monooxygenase (AlkB) is a non-heme diiron integral membrane protein widely found in bacteria capable of growing on medium- to long-chain alkanes as their sole source of carbon and energy (alkanotrophs). Its ability to selectively oxidize alkanes has led to it being the target of numerous biotechnology explorations (McKenna and Coon, 1970; Bosetti et al., 1992; Li et al., 1999; Staijen et al., 2000; van Beilen et al., 2001, 2003; van Beilen and Funhoff, 2005, 2007; Austin and Groves, 2011; Gross et al., 2013; Schrewe et al., 2014; Ladkau et al., 2016; Hoschek et al., 2017; van Nuland et al., 2017). While the three-dimensional structure of the enzyme remains elusive (Xie et al., 2011; Alonso and Roujeinikova, 2012; Alonso et al., 2014), many details of the structure and reaction mechanism have been determined (May and Abbott, 1972; May et al., 1977; May and Katopodis, 1986; van Beilen et al., 1992, 2005; Shanklin et al., 1997; Austin et al., 2000, 2008; Bertrand et al., 2005; Rozhkova-Novosad et al., 2007; Xie et al., 2011; Alonso and Roujeinikova, 2012; Cooper et al., 2012; Alonso et al., 2014). Common to many monooxygenases, the protein starts the catalytic cycle in the diferric state and requires reduction by two electrons to bind and activate oxygen. This Mini Review will focus on the proteins that transfer electrons from the biological electron source [NAD(P)H] to AlkB. It will begin by describing the canonical electron-transfer partners found as part of the OCT operon, which contains all the proteins required to transform alkanes to fatty acids (van Beilen et al., 1994). Fatty acids can be used for metabolism by many bacteria (Jimenez-Diaz et al., 2017). It will then point out the relatively rare occurrence of the OCT operon in sequenced microbial genomes that contain AlkB. Next, it will explore other arrangements of electron-transfer proteins, including two different arrangements in which one or both electron-transfer proteins are found as part of a gene fusion with the hydroxylase. Common themes with other similar proteins will be examined. Finally, the Mini Review will end with a discussion of the possible functional relevance of this diversity of electron-transfer protein arrangements and point to critical unanswered questions for future work.

## The OCT Plasmid, Which Contains the Canonical Electron-Transfer Partners AlkG and AlkT

*P. putida* GPo1 (van Beilen et al., 1994) is the organism whose genetics and biochemistry of liquid alkane metabolism are best characterized. *P. putida* GPo1 harbors an OCT plasmid (van Beilen et al., 1994). This plasmid contains the alkane hydroxylase component, as well as all of the other proteins required to convert alkanes to carboxylic acids (van Beilen et al., 1994). The alkane hydroxylase component consists of an integral membrane protein (AlkB), a rubredoxin (AlkG), and a reductase (AlkT) (van Beilen et al., 1994). The OCT plasmid is a member of the IncP-2 family of plasmids (van Beilen et al., 1994). It can be transmitted to other *Pseudomonads*, although with low frequency. It also encodes proteins for mercury resistance and D-lysine catabolism (van Beilen et al., 1994). In addition to the alkane hydroxylase components, the alkane-oxidizing component of the plasmid, known as *alkBFGHJKL*, encodes another rubredoxin AlkF, which appears to be a duplication of the inactive N–terminal region of AlkG (Kok et al., 1989). No function for AlkF has been found (Kok et al., 1989). AlkJ is a membrane-bound alcohol dehydrogenase with high homology to choline dehydrogenase, another flavin protein that has the potential to direct electrons to the respiratory chain of *Pseudomonas* (Kirmair et al., 2014). AlkH is an aldehyde dehydrogenase, which oxidizes the aldehyde produced by AlkJ to a carboxylic acid (Kok et al., 1989). AlkK is an acyl-CoA synthetase, which activates fatty acids so they can enter the β oxidation cycle (van Beilen et al., 2001; Smits et al., 2002). AlkL is an outer membrane transporter (Julsing et al., 2012; Schrewe et al., 2014). In a slightly different region of the plasmid, two additional genes associated with alkane utilization have been identified (*alkS* and *alkT*). AlkS is a DNA regulator that controls AlkB expression (van Beilen et al., 1994, 2001; Procopio et al., 2013). AlkT is a rubredoxin reductase (Eggink et al., 1990). The easy-to-recognize presence of an entire suite of genes that code for the proteins necessary to convert octane to energy and carbon-building blocks has cemented the notion that AlkB exists solely for this purpose.

Like soluble methane monooxygenase (sMMO; Wang et al., 2014) and cytochrome P450 (CYP; Groves, 1985), AlkB requires two electrons to bind and activate oxygen. NADH is the ultimate electron source for AlkB (van Beilen et al., 2002). NADH reduces the flavin-based rubredoxin reductase AlkT, which in turn reduces AlkG.

**Figure 1:**
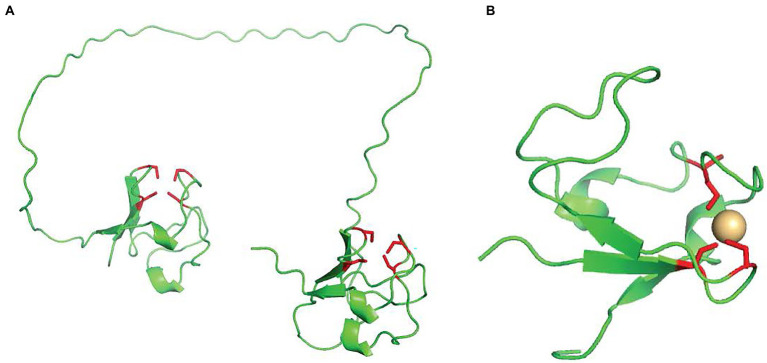
**(A)** The AlphaFold predicted structure of AlkG from *P. putida* GPo1. The cysteines that form the iron–sulfur clusters are colored in red. **(B)** The structure of the single domain rubredoxin from *Pseudomonas aeruginosa*, Again, the cysteines that form the iron–sulfur clusters are colored in red.

AlkG is an unusual rubredoxin, containing two domains, each with an Fe-S cluster (van Beilen et al., 2002). Figure 1A provides an AlphaFold prediction of the structure, which demonstrates the bi-lobal structure (Jumper et al., 2021). The iron ions in the N-terminus (AlkG1) are loosely bound and the rubredoxin in this domain is not essential for activity (van Beilen et al., 2002). The C–terminal rubredoxin AlkG2 is responsible for the redox activity. The two rubredoxins can be distinguished at the sequence level by two CXXCG regions in AlkG2 and CXXCG and CXXCX clusters in AlkG1 (van Beilen et al., 2002).

## Alternatives to the OCT Plasmid and Specifically Alternatives to AlkG and AlkT

The structure of the OCT plasmid is so logical that it often dominates the way people think about alkane oxidation in bacteria in spite of the fact that other arrangements of genes are very common (Smits et al., 2002, 2003). In fact in most alkane-oxidizing strains, the genes involved in alkane degradation are distributed over the genome (Smits et al., 2002).

While *P. putida* GPo1 contains this unusual two-domain AlkG, many other AlkB-containing strains have an isolated AlkG2-like rubredoxin, a structure of which is shown in Figure 1B Organisms that do not have AlkG but have instead an isolated AlkG2-like rubredoxin, often also have a highly conserved AlkG1-like rubredoxin that is not required for activity (Kok et al., 1989; van Beilen et al., 2002). Both a crystal structure (Hagelueken et al., 2007) and an NMR structure (Perry et al., 2001) of a reductase/rubredoxin complex with a single domain rubredoxin exist and point to key amino acids that facilitate contact between the two. The importance of the charged residues on the rubredoxin suggests that oppositely charge residues on AlkB may be important in rubredoxin hydroxylase recognition.

AlkBs have been shown capable of using non-native rubredoxins, although with some limitations (Smits et al., 2002; van Beilen et al., 2002). AlkT can be replaced with other reductases, and even other sources of electrons (Eggink et al., 1987), both *in vivo* (Benson et al., 1977) and *in vitro* (Shanklin et al., 1997; Naing et al., 2013). The ability to use other electron-transfer proteins has simplified efforts to activate AlkB and raises the possibility that non-native electron-transfer partners can be optimized for biotechnology applications (Bernhardt and Urlacher, 2014; Li et al., 2020).

There are other AlkBs that differ even more dramatically in the organization of their electron-transfer partners. A class of AlkBs exists in which the rubredoxin is C–terminally fused to the hydroxylase. These rubredoxin-fused AlkBs appear in a number of different genuses. All of these rubredoxins are single–domain rubredoxins and cluster into a third cluster that is distinct from AlkG2 and AlkG1 (Nie et al., 2011).

It is not yet clear what the functional effect, if any, the fused rubredoxin has on the overall alkane hydroxylase activity. The presence of a rubredoxin-fused AlkB enabled a *Dietzia* strain to grow on very long-chain alkanes, considerably longer than in its absence (Nie et al., 2011). However recent *in vitro* work in our lab comparing the activity of the fused rubredoxin AlkB to a rubredoxin-deletion mutant showed no difference in substrate preference (Williams et al., 2021). Differences in what these two studies measured—in one case growth of an organism and, in the other case, the ability of an enzyme to hydroxylate alkanes of a given chain length *in vitro*—make clear the gaps that remain in linking our understanding of molecular-level processes to an understanding of how these processes are orchestrated at the cellular level, a point we will return to at the end of this Mini Review. Furthermore, the *in vivo* study showed that rubredoxin-fused AlkBs facilitate growth on C32 (Nie et al., 2011), a length of carbon chain outside an easy analytical window for *in vitro* work (Smits et al., 2002). The rubredoxin-fused AlkB of *Prauserella rugosa* NRRL B-2295 could not complement the rubredoxin-deletion mutant, suggesting that this fused rubredoxin was functional ONLY when fused to the hydroxylase (Nie et al., 2011). In contrast, however, the rubredoxin-deletion mutant of AlkB from *D. cinnamea* can accept electrons from an exogenous AlkG (the AlkG from *P. putida* GPo1, in an assay containing NADH, AlkT, and AlkG; Williams et al., 2021).

A bioinformatics analysis (Nie et al., 2014) suggested the existence of a three-domain AlkB in which a ferredoxin reductase and ferredoxin are N–terminally fused to a hydroxylase (ferr_ferrR_AlkB). We recently expressed the protein and showed it to be active (Williams et al., 2021). We also identified the genuses in which this protein can be found (Williams et al., 2021). All current annotations in IMG and NCBI mis-identify the protein as either a fatty acid desaturase or a ferredoxin reductase, neglecting the clear presence of the hydroxylase domain.

## Commonalities With Related Proteins

The significance of the arrangement of electron-transfer partners for AlkB may be better appreciated by comparing them to other related proteins. AlkB is a member of the class III diiron proteins, which also includes the mammalian membrane-spanning fatty acid desaturases and fatty acid hydroxylases (Fox et al., 1994). We will examine the electron-transfer proteins associated with these metalloenzymes as well as cytochromes P450 (CYPs), which perform similar chemistry.

### Fatty Acid Desaturases

Some membrane-bound desaturases use a membrane-bound cytochrome b_5_, which receives electrons from NADH or NADPH through a NADH or NADPH cytochrome oxidoreductase. Some of these desaturases are fused to the cytochrome b_5_ domain, either at the N– or C–terminal end or even as an internal fusion (Sperling and Heinz, 2001; Zäuner et al., 2012). In some fatty acid desaturases, the presence of the fused cytochrome b_5_ domain has been shown to be essential for activity, even *in vivo* (Mitchell and Martin, 1995; Sayanova et al., 1999). No examples of ferredoxin-fused desaturases have been found (Sperling and Heinz, 2001). The most obvious benefit of fusing the electron-transfer partner directly to the enzyme is to improve the rate of electron transfer between two proteins, which is typically slow.

### Fatty Acid Hydroxylases

A fatty acid hydroxylase found in yeast (Fah1p) is found fused to cytochrome b_5_, its electron-transfer partner (Mitchell and Martin, 1997). It is postulated to play a role in hydroxylating very long-chain (C20-C28) fatty acids (Mitchell and Martin, 1997). The mammalian fatty acid hydroxylases also have an N–terminal fused electron-transfer partner (Alderson et al., 2004), although in the crystal structure of this protein, that region was disordered (Zhu et al., 2015). An *in vivo* truncation of the N–terminal cytochrome b_5_ was still functional (Zhu et al., 2015). In humans, mutations in the cytochrome b_5_ domain caused a mild form of hereditary leukodystrophy, in contrast with humans who had mutations in the iron-binding ligands, who had a severe form of the disease (Hama, 2010). This result suggests that the fused electron-transfer partner plays an important, albeit not essential, role in fatty acid hydroxylation.

### Cytochrome P450s (CYPs)

CYP fusion proteins with different architectures are common in bacteria (De Mot and Parret, 2002; Munro et al., 2007). Unlike AlkB, bacterial CYPs usually utilize ferredoxin and ferredoxin reductase for activity (Li et al., 2020). Three bacterial CYP153s (van Beilen et al., 2006), which are the CYPs that oxidize alkanes in the environment, have been shown in a bioinformatics analysis to have an N–terminal CYP domain with a ferredoxin reductase domain and a C–terminal ferredoxin domain (Nie et al., 2014). These genes were found in *G. polyisoprenivorans* VH2, *Gordonia araii* NBRC 100433, and *G. polyisoprenivorans* NBRC 16320. A BLAST search indicates numerous other CYPS with a similar three-domain structure in classes of bacteria known to metabolize alkanes.

CYPs fused to electron-transfer partners are characterized by their high activity, leading to the suggestion that gene fusion of electron-transfer partners increases catalytic activity and decreases uncoupling of reducing agents (Munro et al., 2007). In CYP, where transfer of two sequential electrons is required to generate the catalytically competent reactive intermediate compound 1 (cpd1), it is thought that rapid electron transfer may help to ensure efficient cpd 1 formation (Munro et al., 2007; Li et al., 2020). Fusing the electron-transfer protein to CYP is also thought to aid transcriptional regulation (Munro et al., 2007). There is also evidence that redox partners can alter the profile of reaction products, perhaps by direct protein–protein interactions that influence the active site conformation (Li et al., 2020). Fusion proteins may lock in one particular activity, while having separate electron-transfer proteins may provide more versatility to CYPs *in vivo* (Li et al., 2020).

Intriguingly, however, the ability to deliver electrons rapidly may lead to an increase in the generation of overoxidation products. Fusion proteins have been shown to catalyze more overoxidation of products (Boddupalli et al., 1992; van Beilen et al., 2003).

## Discussion

One point that emerges from this effort to understand the functional role of AlkB’s diverse electron-transfer partners is the disconnections that exist between the different methods of investigation. Some groups have studied the *in vivo* effects of various gene deletions. Other groups have studied what is biochemically feasible *in vitro* at various stages of protein purification. Alkane metabolism requires the transport of substrates from outside the organism to the active site, as well as the ability to use the product to extract energy and carbon. Therefore, studying growth on alkanes encapsulates multiple distinct biochemical processes. Biochemical studies can reveal what chemistry a protein is capable of, but as we and others have shown, the membrane milieu can change the chemistry of an enzyme such that experiments done on highly purified enzymes might not capture even the fundamental biochemistry *in vivo* (van Beilen et al., 1994; Ro et al., 2018).

In spite of more than 50 years of work on microbial alkane metabolism, many questions remain. Some of the questions are brought into sharper focus by the wealth of genomic and metagenomic data that exists. Below, we outline a few of the pressing questions and suggest approaches to answer them.

We remain puzzled by the functional role, if any, of the second rubredoxin in the canonical AlkG. We are also puzzled by the presence of AlkF and other AlkG1-like rubredoxins that occur frequently in the genome of alkane-oxidizing organisms. The highly conserved sequence suggests a functional role, although we and others have not been successful in endeavors to identify that role. Effector proteins have been shown to be important in other hydroxlases (Lountos et al., 2005; Mitić et al., 2008), where their effects are often subtle. More *in vitro* studies may shed light on conditions where AlkF impacts the chemistry of AlkB. Functional roles for AlkF might emerge from additional whole cells screens.

We see several possible roles for fusion of electron-transfer partners directly to AlkB. The most obvious role is that electron transfer rates in fusion proteins are much faster than in systems where the electron-transfer proteins need to find their redox partners in space, facilitating alkane oxidation. Fusion of the electron-transfer partners also simplifies regulation of alkane oxidation as only one protein needs to be upregulated in response to the presence of alkanes in this system. Commonly, in systems where the redox partners are not fused, the electron-transfer proteins are differentially regulated (Hagelueken et al., 2007). They may occur on different parts of the chromosome and possibly serve as electron-transfer proteins for other enzymes. Another suggestion is that longer chain alkanes interact with more sites within AlkB, which disrupt rubredoxin binding, thereby necessitating the sort of pre-formed structures that could exist with a fusion protein (Lee et al., 1996; Nie et al., 2011). Intriguingly, prediction of the three-dimensional structure of ferr_ferrR_AlkB by AlphaFold (Jumper et al., 2021) shows all of the key redox active partners in close proximity, even in the absence of metal binding (which AlphaFold cannot, at this point, predict).

It is possible that the presence of electron-transfer proteins fused to the hydroxylase changes the chemistry of the enzyme. It is not yet clear whether AlkB substrates, once transformed to products, exit the substrate channel in the same way they entered it. If so, then the covalent association of electron-transfer proteins could alter the channel structure in ways that could slow product release. AlkBs have been shown capable of oxidizing alkanes to fatty acids; perhaps the presence of the fused rubredoxin slows the rate of product release and facilitates the accumulation of neutral lipids (van Beilen et al., 2003), as has been suggested for fused CYPs (Boddupalli et al., 1992).

We also note that ferr_ferrR_AlkB does not epoxidize alkenes, unlike all of the other AlkBs that have been characterized (Williams et al., 2021). Alkene epoxidation requires some substrate mobility to allow the diiron active site to access the terminal carbon for initial O-atom insertion and then the adjacent carbon to form the epoxide ring. While much work remains to be done to study this phenomenon, it suggests a structural change resulting from the fusion. Understanding the functional impacts of electron-transfer partners is therefore important for biotechnology applications of AlkB.

To what extent, if any, is the presence of other OCT operon genes indicative of alkane mineralization? And, conversely, are *alkBs* functional when they are not near genes encoding other proteins required for alkane oxidation? If so, what are their biological roles? While most *alkB*s are upregulated by the presence of alkanes, not all are (Nie et al., 2011), which again might suggest a functional role outside of alkane oxidation. It has been suggested that the OCT plasmid might be valuable for organisms that have few carbon sources outside of alkanes, while organisms—like most of the pathogens that encode an active AlkB—that can use a variety of carbon sources instead activate AlkB from electron-transfer partners found elsewhere on the genome and hence do not find the OCT plasmid to be adventageous (Smits et al., 2002). We (Williams et al., 2021) and others (Koch et al., 1991) have noted a connection between AlkB and rhamnolipids, but the nature of this connection is not clear.

## Future Directions

There are many future directions that an interest in the electron-transfer proteins associated with AlkB may take.

More structural information is needed on the nature of the protein–protein interactions that mediate electron transfer. A three-dimensional structure of AlkB would be the starting point for this work. A structure would make it easier to identify possible sites of association for the rubredoxin. We are hopeful that a non-disordered structure of the two- and three-domain AlkB-fusion proteins will also provide information about the structural determinants of electron transfer. We are carrying out mutational studies on both rubredoxins and AlkB to try to pinpoint residues that are critical in protein–protein interactions and electron transfer.

There is interest in making synthetic “self-sufficient” monooxygenases by using tools from molecular biology to fuse electron-transfer partners directly to the catalyst. These synthetic fusion proteins could have more highly specialized functions, simpler transcriptional regulation, and greater ease in biotechnological uses. The ones that already exist in nature that we point to in this Mini Review might be useful “as-is” in various synthetic processes, or after some directed or random evolutionary pressure has been applied to them to alter their substrate scope.

More experiments are required to probe whether AlkBs have functions other than simply activating alkanes for degradation, for example, whether they play any biosynthetic roles.

In conclusion, AlkB is a critical enzyme in the global carbon cycle, whose activity requires that the active site be reduced. Awareness of the unusual structure of AlkG from *P. putida* GPo1 has existed for many years. However, the role of the N–terminal rubredoxin domain in some AlkGs is still unknown. The role of AlkF is also unknown. The dramatic increase in genomic information makes it possible to more broadly interrogate gene neighborhoods, a project which has made clear that the OCT operon represents a minority location for *alkB* genes. Bioinformatic studies have made visible the frequency of two- and three-domain AlkBs, work we have followed up with by biochemically characterizing those proteins. More work remains to be done to connect their biochemistry to their functions in biology and the environment.

## Author Contributions

RNA and SCW both wrote and revised the manuscript. All authors contributed to the article and approved the submitted version.

## Funding

The work was funded by NIH RO1GM130989 (to RNA).

## Conflict of Interest

The authors declare that the research was conducted in the absence of any commercial or financial relationships that could be construed as a potential conflict of interest.

## Publisher’s Note

All claims expressed in this article are solely those of the authors and do not necessarily represent those of their affiliated organizations, or those of the publisher, the editors and the reviewers. Any product that may be evaluated in this article, or claim that may be made by its manufacturer, is not guaranteed or endorsed by the publisher.
